# Supplementary material for: The immune factors driving DNA methylation variation in human blood
Source: Nat Commun. 2022 Oct 6;13:5895. doi: 10.1038/s41467-022-33511-6 (PMC9537159; doi:10.1038/s41467-022-33511-6)
Supplement: Supplementary file 3 — Description of Additional Supplementary Files [file 41467_2022_33511_MOESM3_ESM.pdf]

Description of Additional Supplementary Files

File Name: Supplementary Data 1
Description: Candidate intrinsic and environmental factors tested for association with the blood DNA methylome of adults

File Name: Supplementary Data 2
Description: Number of CpG sites associated with cell proportion contrasts and definitions of contrasts by sequential binary partition

File Name: Supplementary Data 3
Description: CpG sites significantly associated with two interacting variables

File Name: Supplementary Data 4
Description: Significant enrichments of variable-associated CpG sites in binding sites for transcription factors (TFs)

File Name: Supplementary Data 5
Description: Significant gene ontology enrichments for genes close to variable-associated CpG sites

File Name: Supplementary Data 6
Description: Summary statistics for significant remote-effect meQTLs

File Name: Supplementary Data 7
Description: Proportions of variance explained by intrinsic factors, exposures, cell composition and local SNPs for the 10,000 CpG sites with the most explained variance
