# Supplementary material for: The immune factors driving DNA methylation variation in human blood
Source: Nat Commun. 2022 Oct 6;13:5895. doi: 10.1038/s41467-022-33511-6 (PMC9537159; doi:10.1038/s41467-022-33511-6)
Supplement: Supplementary file 11 — Reporting Summary [file 41467_2022_33511_MOESM11_ESM.pdf]

nature research

Corresponding author(s): Lluis QUINTANA-MURCI

Last updated by author(s): Aug 24, 2022

Reporting Summary

Nature Research wishes to improve the reproducibility of the work that we publish. This form provides structure for consistency and transparency in reporting. For further information on Nature Research policies, see our Editorial Policies and the Editorial Policy Checklist.

Statistics

For all statistical analyses, confirm that the following items are present in the figure legend, table legend, main text, or Methods section.

n/a	Confirmed	
☐	☒	The exact sample size (*n*) for each experimental group/condition, given as a discrete number and unit of measurement
☐	☒	A statement on whether measurements were taken from distinct samples or whether the same sample was measured repeatedly
☐	☒	The statistical test(s) used AND whether they are one- or two-sided<br>*Only common tests should be described solely by name; describe more complex techniques in the Methods section.*
☐	☒	A description of all covariates tested
☐	☒	A description of any assumptions or corrections, such as tests of normality and adjustment for multiple comparisons
☐	☒	A full description of the statistical parameters including central tendency (e.g. means) or other basic estimates (e.g. regression coefficient) AND variation (e.g. standard deviation) or associated estimates of uncertainty (e.g. confidence intervals)
☐	☒	For null hypothesis testing, the test statistic (e.g. *F*, *t*, *r*) with confidence intervals, effect sizes, degrees of freedom and *P* value noted<br>*Give P values as exact values whenever suitable.*
☒	☐	For Bayesian analysis, information on the choice of priors and Markov chain Monte Carlo settings
☐	☒	For hierarchical and complex designs, identification of the appropriate level for tests and full reporting of outcomes
☐	☒	Estimates of effect sizes (e.g. Cohen's *d*, Pearson's *r*), indicating how they were calculated

Our web collection on statistics for biologists contains articles on many of the points above.

Software and code

Policy information about availability of computer code

Data collection	R-4.1.0 packages: minfi (v.1.40.0); R-3.6.2 packages: irlba (v.2.3.5), sva (v.3.34.0)
Data analysis	R-4.1.0 packages: missForest (v.1.4), FlowSorted.BloodExtended.EPIC (v.0.9.2), FlowSorted.Blood.EPIC (v.1.12.1); R-3.6.2 packages: lme4 (v.1.1-29), MatrixEQTL (v.2.3), robCompositions (v.2.3.1), sandwich (v.3.0-2), pbkrtest (v.0.5.1), gamlss (v.5.4-3), missMethyl (v.1.20.4), dirmult (v.0.1.3-5), glmnet (v.4.1-2); PLINK (v.1.9); other custom-generated scripts are deposited on GitHub (https://github.com/JacobBergstedt/MIMETH)

For manuscripts utilizing custom algorithms or software that are central to the research but not yet described in published literature, software must be made available to editors and reviewers. We strongly encourage code deposition in a community repository (e.g. GitHub). See the Nature Research guidelines for submitting code & software for further information.

Data

Policy information about availability of data

All manuscripts must include a data availability statement. This statement should provide the following information, where applicable:

- Accession codes, unique identifiers, or web links for publicly available datasets
- A list of figures that have associated raw data
- A description of any restrictions on data availability

The Infinium MethylationEPIC raw and processed data generated in this study have been deposited in the Institut Pasteur data repository, OWEY, which can be accessed via the following link: https://dataset.owey.io/doi/10.48802/owey.f83a-1042. All association statistics obtained in this study (i.e., the 141 EWAS and interaction models, local meQTL mapping) can be explored and downloaded from the web browser http://mimeth.pasteur.fr/. The SNP array data can be accessed in the European Genome-Phenome Archive (EGA) with the accession code EGAS00001002460. All Milieu Intérieur datasets can be accessed by submitting a data

access request to milieuinterieurdac@pasteur.fr, the Milieu Intérieur data access committee, which grants data access if the request is consistent with the informed consent provided by Milieu Intérieur participants. Requests are reviewed every month by the committee.

Field-specific reporting

Please select the one below that is the best fit for your research. If you are not sure, read the appropriate sections before making your selection.

☒ Life sciences ☐ Behavioural & social sciences ☐ Ecological, evolutionary & environmental sciences

For a reference copy of the document with all sections, see nature.com/documents/nr-reporting-summary-flat.pdf

Life sciences study design

All studies must disclose on these points even when the disclosure is negative.

Sample size	The sample size is the total size of the Milieu Intérieur cohort (n = 1000), for which extensive phenotype data has been collected. Using simulations, we found that we have 95% power to detect a meQTL variant with a medium effect of 0.6 phenotype standard deviation (Patin et al., Nat Immunol 2018).
Data exclusions	Donors were required to have no history or evidence of severe/chronic/recurrent pathological conditions, neurological or psychiatric disorders, alcohol abuse, recent use of illicit drugs, recent vaccine administration, and recent use of immune modulatory agents. To avoid the influence of hormonal fluctuations in women, pregnant and peri-menopausal women were not included. To avoid genetic stratification in the study population, the recruitment of donors was restricted to individuals whose parents and grandparents were born in Metropolitan France. Criteria are detailed in a separate publication (Thomas et al., Clin Immunol 2015).
Replication	DNA methylation levels were measured with the Illumina MethylationEPIC array and were not replicated. Previous studies have demonstrated that DNA methylation levels measured by the MethylationEPIC array are highly correlated between technical replicates (Spearman rank correlation ρ=0.993; Pidsley et al., Genome Biol 2016).
Randomization	The study is not a randomized controlled trial. It is an observational study that aims to identify environmental exposures and genetic factors affecting the blood DNA methylome of healthy adults.
Blinding	No experiments, whether blinded or not, were conducted on research participants. The study is an observational study that aims to identify environmental exposures and genetic factors affecting the blood DNA methylome of healthy adults.

Reporting for specific materials, systems and methods

We require information from authors about some types of materials, experimental systems and methods used in many studies. Here, indicate whether each material, system or method listed is relevant to your study. If you are not sure if a list item applies to your research, read the appropriate section before selecting a response.

Materials & experimental systems

n/a	Involved in the study	
☒	☐	Antibodies
☒	☐	Eukaryotic cell lines
☒	☐	Palaeontology and archaeology
☒	☐	Animals and other organisms
☐	☒	Human research participants
☐	☒	Clinical data
☒	☐	Dual use research of concern

Methods

n/a	Involved in the study	
☒	☐	ChIP-seq
☐	☒	Flow cytometry
☒	☐	MRI-based neuroimaging

Human research participants

Policy information about studies involving human research participants

Population characteristics	The 1,000 healthy donors of the Milieu Intérieur cohort include 100 women and 100 men from each decade of life, between 20 and 69 years of age. Detailed cohort characteristics (i.e., 141 variables) can be found in Supplementary Data 1.
Recruitment	A pre-existing donor database composed of ~110,000 donors was used for pre-screening potential participants in accordance with the study criteria. Additional advertising and website recruitment campaigns were launched in order to complete strata not sufficiently represented in the donor database. Eligibility was assessed by telephone interview and confirmed during a preliminary information meeting about the objectives of the research. Interested participants that met pre-screening criteria returned for the enrollment visit. During this visit, eligibility criteria were assessed in two stages: first, based on demographical data and clinical examination; and second, by analysis of blood and urine samples that were sent for clinical laboratory testing (Thomas et al., Clin Immunol 2015). We found that the cohort is representative of the general adult healthy population, except that it presents a ~10% higher unemployment rate and a higher average educational level

	(Thomas et al., Clin Immunol 2015). This recruitment bias is not expected to affect the study results, as DNA methylation levels are not associated with socio-economic status in the Milieu Intérieur cohort (Supplementary Data 1).
Ethics oversight	The study is sponsored by the Institut Pasteur (Pasteur ID-RCB Number: 2012-A00238-35) and was conducted as a single center study without any investigational product. The Milieu Intérieur clinical study was approved by the Comité de Protection des Personnes — Ouest 6 (Committee for the protection of persons) on June 13, 2012 and by the French Agence Nationale de Sécurité du Médicament (ANSM) on June 22, 2012. The samples and data used in this study were formally established as the Milieu Intérieur biocollection (study# NCT03905993), with approvals by the Comité de Protection des Personnes – Sud Méditerranée and the Commission nationale de l'informatique et des libertés (CNIL) on April 11, 2018.

Note that full information on the approval of the study protocol must also be provided in the manuscript.

Clinical data

Policy information about clinical studies

All manuscripts should comply with the ICMJE guidelines for publication of clinical research and a completed CONSORT checklist must be included with all submissions.

Clinical trial registration	NCT03905993
Study protocol	The study protocol is described in Thomas et al., Clin Immunol 2015.
Data collection	Whole blood samples were collected from the 1,000 Milieu Intérieur healthy, fasting donors, every working day from 8AM to 11AM, from September 2012 to August 2013, in the offices of the BioTrial research institute (Rennes, France). Tracking procedures were established in order to ensure delivery to Institut Pasteur (Paris, France) within 6 hours of blood draw, at a temperature between 18° C and 25°C. Upon receipt, samples were kept at room temperature until DNA extraction. DNA was extracted using the Nucleon BACC3 genomic DNA extraction kit (Cytiva, Massachusetts, USA). High-quality genomic DNA was obtained for 978 out of the 1,000 donors.
Outcomes	The aim of the study is to determine the non-genetic and genetic determinants for epigenetic variation in human healthy adults. We defined as primary outcomes the 5mC levels of hundred thousands of CpG sites, because they can be obtained reproducibly and cost effectively for large cohorts using beadchips. Extracted genomic DNA was treated with the EZ DNA Methylation Kit (Zymo Research, California, USA). Bisulfite-converted DNA was applied to the Infinium MethylationEPIC BeadChip (Illumina, California, USA), using the manufacturer's standard conditions. The MethylationEPIC BeadChip measures 5mC levels at 866,836 CpG sites in the human genome.

Flow Cytometry

Plots

Confirm that:

- ☐ The axis labels state the marker and fluorochrome used (e.g. CD4-FITC).
- ☐ The axis scales are clearly visible. Include numbers along axes only for bottom left plot of group (a 'group' is an analysis of identical markers).
- ☐ All plots are contour plots with outliers or pseudocolor plots.
- ☐ A numerical value for number of cells or percentage (with statistics) is provided.

Methodology

Sample preparation	Flow cytometry was performed on whole blood, collected using Li-heparin as an anti-coagulant and maintained at room temperature (18–25°C) until processing. The cells were stained using commercially available monoclonal antibodies, according to the operating procedure developed as described in Hasan et al., Clin Immunol 2015. Red cells were lysed to optimize the staining protocol. Fc-receptor blocking antibodies were used (eBioscience ref. 14-9161). Dead cells were excluded using the Fixable Viability Dye (FVD) eFluor 506 (eBioscience, ref. 65-0866).
Instrument	MACSQuant Analyzer 10 benchtop flow cytometer (Miltenyi Biotec).
Software	FlowJo software version V10.6.1
Cell population abundance	The proportions of 16 blood cell subsets were used to quantify blood cell composition. There respective abundance can be found in Patin*, Hasan*, Bergstedt* et al., Nat Immunol 2018.
Gating strategy	Gating strategies for the 10 eight-color panels are described in full detail in Patin*, Hasan*, Bergstedt* et al., Nat Immunol 2018.

☐ Tick this box to confirm that a figure exemplifying the gating strategy is provided in the Supplementary Information.
